# Adding a low-concentration sciatic nerve block to total knee arthroplasty in patients susceptible to the adverse effects of non-steroidal anti-inflammatory drugs (NSAIDs): a randomized controlled trial

**DOI:** 10.1186/s12871-021-01491-7

**Published:** 2021-11-13

**Authors:** Busara Sirivanasandha, Kulwadee Sutthivaiyakit, Thippatai Kerdchan, Suppachai Poolsuppasit, Suwimon Tangwiwat, Pathom Halilamien

**Affiliations:** Department of Anesthesiology, Faculty of Medicine Siriraj Hospital, Mahidol University, 2 Wanglang Road, Bangkok Noi, Bangkok, 10700 Thailand

**Keywords:** Adverse effects of NSAIDs, Popliteal-sciatic nerve block (SNB), Dexamethasone, Total knee arthroplasty (TKA), Adductor canal blocks, Pain management

## Abstract

**Background:**

This study compared the effects of adductor canal blocks with those of a low concentration of popliteal-sciatic nerve block (SNB) and dexamethasone as an adjunctive technique for total knee arthroplasties (TKA) in patients susceptible to the adverse effects of NSAIDs.

**Methods:**

A prospective, double-blinded, randomized controlled trial was performed in 50 patients susceptible to the adverse effects of NSAIDs undergoing unilateral TKAs. All patients received spinal anesthesia, adductor canal blocks, and periarticular infiltration. The 25 patients in the intervention group received SNB (0.125% bupivacaine [20 ml] and dexamethasone [5 mg]).

**Results:**

The SNB group significantly had lower median resting pain scores at 6, 12, and 18 h: the control group, 1 (0–4.5), 3 (0–5), and 3 (2–5); the intervention group, 0 (0–0), 0 (0–3), and 1 (0–3); *p*-values, 0.012, 0.021, and 0.010, respectively. Movement-evoked pain scores at 6, 12, and 18 h were also lower: control group, 3 (0–5.5), 5 (2.5–6.5), and 7 (4–9); intervention group, 0 (0–1.5), 2 (0–4), and 3 (2–5); *p*-values, 0.019, 0.005, and 0.001, respectively. There were no differences in motor function. Moreover, the mean morphine consumption 24 h was also reduced in the SNB group: control group, 3.80 ± 2.48 mg; intervention group, 1.96 ± 2 mg; *p*-value, 0.005.

**Conclusion:**

For patients susceptible to the adverse effects of NSAIDs, a low concentration of SNB and dexamethasone is an effective adjunctive technique for early postoperative pain control (especially on movement) following TKAs, without an increase in motor weakness.

**Trial registration:**

ClinicalTrials.gov, NCT03486548, Registered 3 April 2018.

## Background

Total knee arthroplasty (TKA), one of the most common surgeries, causes moderate to severe postoperative pain. Multimodal analgesia is currently the standard treatment for acute postoperative pain control, and it aims to reduce the usage of opioids. The absence of opioid side effects promotes early ambulation, hastens postoperative recovery, decreases the incidence of postoperative complications, and improves patient satisfaction [1].

A peripheral nerve block is a popular technique for acute postoperative pain control after TKA. The sensory nerves that supply the anterior side of the knee originate from branches of the femoral nerve, the lateral femoral cutaneous nerve, and the common peroneal nerve. In comparison, the posterior side of the knee is supplied by the popliteal nerve plexus, which derives from the tibial nerve, and branches of the obturator nerve [2, 3]. While postoperative knee pain can be reduced with either an adductor canal block or a femoral nerve block, the former has a lower incidence of quadriceps muscle weakness [4–6].

At our institute, multimodal analgesia is currently utilized for postoperative pain control after TKA. Depending on the judgement of the attending anesthesiologist, the multimodal analgesia comprises a varying combination of spinal anesthesia, adductor canal block, periarticular infiltration, postoperative multimodal medication, and systemic nonsteroidal anti-inflammatory drugs (NSAIDs). The study by Gwam et al. reported an average pain score of 4.94 (range 0–9) for TKA patients whose postoperative pain was being managed with a combination of an adductor canal block and periarticular infiltration [7]. Moreover, periarticular infiltration may only reduce pain for 6 to 12 h after surgery [8]. Therefore, NSAIDs are the main agents used to relieve pain and reduce opioid consumption.

Patients susceptible to the adverse effects of NSAIDs—such as patients with chronic kidney disease, coronary artery disease, gastric ulcer, a history of stroke, or advanced age—have a limited ability to use systemic NSAIDs postoperatively but can be safe for a local infiltration with NSAIDs has the local rather than systemic effect [9, 10]. Without the opioid-sparing effects of NSAIDs [11–13], patients in this group may suffer from worse postoperative pain levels than previously reported by Gwam et al.

A sciatic nerve block (SNB) is considered to reduce posterior knee pain [14, 15]. It may cause hamstring muscle weakness or foot drop, resulting in delayed postoperative ambulation [16]. A low concentration of local anesthetic may reduce the risk of a motor block, but it may also affect its analgesic duration. However, a systematic review and meta-analysis reported that adding dexamethasone to the local anesthetic drug injected into nerve areas can increase the analgesic duration of the local anesthetic without causing severe side effects [17, 18].

The primary purpose of this study was to evaluate the benefit of overall postoperative pain control by adding a combination of a low concentration of popliteal-SNB and dexamethasone as an adjunctive technique for TKA in patients susceptible to the adverse effects of NSAIDs. In addition, it compared their effects with those of an adductor canal block (ACB) and periarticular infiltration analgesia (PIA) for such patients.

## Methods

### Study design and population

Prior to commencement, this single-center, prospective, double-blinded, randomized controlled trial was approved by the Siriraj Institutional Review Board (COA no. Si123/2018) and registered at ClinicalTrials.gov, NCT03486548, Registered 3 April 2018, https://clinicaltrials.gov/ct2/show/NCT03486548. The investigation was conducted on patients aged 50–85 years; had American Society of Anesthesiologists physical statuses of I–III; and were scheduled to undergo elective, unilateral, primary TKA at Siriraj Hospital May 2018–December 2019. The inclusion criteria were patients susceptible to the adverse effects of NSAIDs: history of chronic kidney disease (estimated glomerular filtration rate < 50 ml/min/1.73 m^2^), coronary artery disease, cerebrovascular disease, gastric ulcer, or an allergy to NSAIDs other than ketorolac. The exclusion criteria were a bodyweight of < 45 kg; allergy to bupivacaine, ketorolac, or dexamethasone; uncontrolled diabetes; contraindication to regional anesthesia; cognitive impairment; preexisting neuropathy; a neurological deficit in the lower extremities; and preexisting pain requiring the chronic use of oral morphine (20 mg/day or equivalent). Patients who declined to participate were also excluded.

### Randomization and blinding

The patients (*n* = 50) were randomized into an intervention group and a control group in blocks of four. This was achieved by using the sealed-envelope technique and computer-generated randomization lists sourced from an internet-based application (www.randomization.com). In the intervention group (*n* = 25), the patients received an SNB with an infusion of 0.125% bupivacaine (20 ml) and dexamethasone (5 mg). As to the control group (*n* = 25), the patients received only a subcutaneous injection of 1% xylocaine (1 ml) in the popliteal area without an SNB. The patients and assessors were blinded to the treatment allocations.

### Anesthetic protocol

After being briefed on the research objectives and the experimental methods, each eligible patient signed a consent document on their respective day of admission. Two hours before surgery, each patient was given an oral premedication of paracetamol (1000 mg). Following standard anesthetic monitoring (pulse oximetry, non-invasive blood pressure monitoring, and electrocardiogram), the patients were sedated using intravenous fentanyl (25–50 mcg) and midazolam (0.5–1 mg) before being administered oxygen through a nasal cannula (3 L/min).

For both groups, an anesthesiologist specializing in regional anesthesia performed an ultrasound-guided ACB in the popliteal area, using 15 ml of 0.33% bupivacaine. All patients were then placed in the lateral decubitus position. Both the control- and intervention-group patients were administered a subcutaneous local infiltration of 1% xylocaine (1 ml). However, the intervention-group patients subsequently also received an ultrasound-guided SNB (the popliteal approach); for that procedure, a mix of 0.125% bupivacaine (20 ml) and dexamethasone (5 mg) was used.

In the operating room, the patients received 2.0 ml of spinal anesthesia with isobaric bupivacaine and an intravenous propofol infusion (25 to 50 mcg/kg/min) to achieve a mild to moderate sedation. At the end of the operation, all patients received PIA with 0.25% bupivacaine (40 ml) and ketorolac (30 mg). During the first three postoperative days, the standard oral postoperative regimen was followed for all patients: paracetamol (1000 mg every 6 h), gabapentin (300 mg before bedtime), and intravenous morphine for pain, as needed (1 mg/hour).

### Outcomes and measurement

The primary outcome was the effects of using a combination of a low concentration of popliteal-SNB and dexamethasone as an adjunctive technique for TKA in patients susceptible to the adverse effects of NSAIDs on their pain scores during the first 24 postoperative hours. Postoperatively, the pain scores and motor function were determined by only one assessor, who was blinded to the group allocations. At 6, 12, 18, and 24 h after surgery, we assessed the postoperative pain scores at the anterior and posterior aspects of the knee while at rest and with motion (45 degrees of knee flexion). At the same times, the function of the tibialis anterior muscle was assessed and graded into 3 categories: 0 (normal); 1 (dorsiflexion, but less powerful than the untreated side); and 2 = no strength to flex the ankle. Pain scores during physiotherapy were also recorded.

### Sample size

Drawing upon data relating to adductor canal blocks and multimodal periarticular analgesia from work by Gwam et al. [7], a prospective power analysis revealed that 50 patients provided a 90% chance (power) to detect a reduction in visual-analog-scale score of 1.94 out of 10 during the first 24 h after surgery. The value of 1.94 represented a reduction from a moderate level of pain (scores 4–6) to a mild level (scores 1–3), e.g., from 4.94 in the control group to 3.00 in the SNB group. The type I error was 0.05 by an F-test; the standard deviation was not greater than 1.86 of the means; and a correction of the values for non-normal distribution was assumed.

### Statistical analysis

All data were analyzed using IBM SPSS Statistics for Windows (version 24.0; IBM Corp., Armonk, NY, USA). The data were tested for normal distribution using histograms. Continuous data were presented as means and standard deviations (SD) or as medians and interquartile ranges (IQR), whereas categorical data were reported as numbers and percentages. A comparative analysis of the data of the 2 groups used the chi-squared test for group data, the unpaired t-test for continuous data with normal distribution, and the Mann–Whitney U test for continuous data with an abnormal distribution.

## Results

**Fig. 1:**
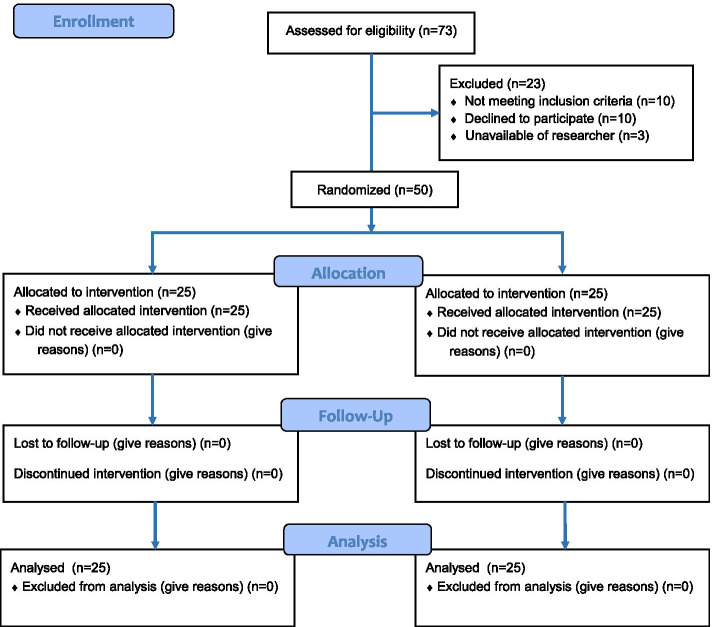
CONSORT 2010 flow diagram

In all, 63 patients who met the inclusion criteria were recruited. However, ten declined the invitation to join the study, while another three were not enrolled because of the unavailability of the researcher (Fig. 1 CONSORT 2010 flow diagram).

The 50 enrolled patients were divided into an intervention group and a control group (25 patients each). No significant differences were found in the age and sex distributions, body mass indexes, estimated glomerular filtration rates, underlying diseases, preoperative pain scores, and surgical and tourniquet times of the 2 groups (Table 1).

**Table 1:** Demographic data

	Control (***n*** = 25)	SNB (***n*** = 25)	***P***-value
Female (n, %)	18 (72%)	19 (76%)	0.747
Age (mean ± SD)	73.88 ± 7.76	73.72 ± 6.94	0.939
BMI (mean ± SD)	28.51 ± 5.43	26.28 ± 3.00	0.080
Underlying disease			
CKD (n, %)	17 (68%)	16 (64%)	0.765
DM (n, %)	11 (44%)	13 (52%)	0.571
HT (n, %)	21 (84%)	24 (96%)	0.349
IHD (n, %)	7 (28%)	7 (28%)	1.000
Stroke (n, %)	8 (32%)	6 (24%)	0.529
eGFR (mean ± SD)	48.90 ± 21.91	52.94 ± 22.13	0.520
Preoperative pain (median [IQR])	3 (2–5)	4 (3–4.5)	0.835
Operative time (min) (mean ± SD)	90 ± 21	95 ± 31	0.462
Tourniquet time (min) (mean ± SD)	85 ± 17	84 ± 24	0.850

*BMI* body mass index, *CKD* chronic kidney disease, *Control* control group, *Cr* creatinine, *DM* diabetes mellitus, *eGFR* glomerular filtration rate, *HT* hypertension, *IHD* ischemic heart disease, *SNB* popliteal-sciatic nerve block group

As to the postoperative pain scores of the groups (Table 2), there were significant differences in their pain scores at rest, pain scores on movement, and anterior-knee pain scores during the first 18 h after the surgery. The median (IQR) pain scores at rest at the 6-, 12-, and 18-h timepoints were 1 (0–4.5), 3 (0–5), and 3 (2–5) for the control group; and 0 (0–0), 0 (0–3), and 1 (0–3) for the intervention group, respectively (*p*-values = 0.012, 0.021, and 0.010). The median (IQR) pain scores on movement at 6, 12, and 18 h were 3 (0–5.5), 5 (2.5–6.5), and 7 (4–9) for the control group, and 0 (0–1.5), 2 (0–4) and 3 (2–5) for the intervention group (*p*-values = 0.019, 0.005, and 0.001). The median (IQR) anterior-knee pain scores at 6, 12, and 18 h were 2 (0–4.5), 2 (0–4), and 3 (0.5–5) for the control group, and 0 (0–0), 0 (0–3), and 1 (0–3) for the intervention group (*p*-values = 0.002, 0.032, and 0.015).

**Table 2:** Postoperative results

	Control (***n*** = 25)		SNB (***n*** = 25)		***P***-value
	Median	(25–75)	Median	(25–75)	
**Pain at rest** (NRS; 0–10)					
6 h	1	0–4.5	0	0–0	0.012*
12 h	3	0–5	0	0–3	0.021*
18 h	3	2–5	1	0–3	0.010*
24 h	3	1–5	2	1–3.5	0.187
**Pain on movement** (NRS; 0–10)					
6 h	3	0–5.5	0	0–1.5	0.019*
12 h	5	2.5–6.5	2	0–4	0.005*
18 h	7	4–9	3	2–5	0.001*
24 h	5	4.5–8	5	2.5–7	0.169
**Anterior-knee pain** (NRS; 0–10)					
6 h	2	0–4.5	0	0–0	0.002*
12 h	2	0–4	0	0–3	0.032*
18 h	3	0.5–5	1	0–3	0.015*
24 h	3	1.5–5	2	0–2.5	0.028*
**Posterior-knee pain** (NRS; 0–10)					
6 h	0	0–0	0	0–0	0.370
12 h	0	0–2.5	0	0–0	0.038*
18 h	0	0–2	0	0–0	0.129
24 h	0	0–2	0	0–1	0.666
**Motor weakness** (n, %)					
6 h	4	16%	6	24%	0.615
12 h	2	8%	2	8%	1.000
18 h	1	4%	1	4%	1.000
24 h	1	4%	0	0%	1.000
**Pain on physiotherapy** (NRS; 0–10)					
24 h	6	5–8	5	3–7	0.083
48 h	3.5	2.3–6.8	3.5	2–5	0.478
**Patient satisfaction score** (0–10)	10	8–10	10	10–10	0.085
**Total morphine in 24 h** (mg)	4	2–8	2	0–2.5	0.005*

*(25–75)* interquartile range 25, 75, *Control* control group, *NRS* numeric rating scale, *SNB* popliteal-sciatic nerve block group, * *P*-value < 0.05

In contrast, a significant difference in the posterior pain scores was found only at the 12-h timepoint: the median (IQR) were 0 (0–2.5) and 0 (0–0) for the control and intervention groups, respectively (*p*-value = 0.038). There were also no significant differences in the motor function of the tibialis anterior muscle, the pain scores recorded during physiotherapy within 24 h and 48 h, nor the levels of patient satisfaction of the 2 groups.

The total morphine consumption of the 2 groups differed significantly during the first 24 h after the TKAs (Table 2). The median (IQR) total morphine consumptions of the control and intervention groups were 4 (2–8) mg and 2 (0–2.5) mg (*p*-value = 0.005). Nevertheless, there was no statistical difference in their hospital lengths of stay, with mean ± SD values of 132 ± 25 h and 128 ± 25 h for the control and intervention groups, respectively (*p*-value = 0.591).

## Discussion

Multimodal analgesia is currently a standard treatment for pain control. Nonsteroidal anti-inflammatory drugs play an essential role in reducing opioid consumption and acute postoperative pain management in orthopedic surgeries, including TKA. However, some patients are subject to the adverse effects of NSAIDs when prescribed systemically due to their potential side effects. Therefore, systemic NSAIDs are usually avoided in these patients, leading to inadequate pain control postoperatively. Our study aimed to focus on postoperative pain control following TKA in these challenging patients by adding SNB to ACB and PIA blocks. To our knowledge, this is the first study in TKA focusing on the NSAIDs susceptible patients.

In this study, pain scores (both at rest and on movement), as well as 24-h morphine consumption, were significantly lower in the SNB group compared to the control group. In the previous studies, SNB was an effective rescue block in patients with severe posterior knee pain [9, 19]. However, adopting such a rescue block might be problematic at wards with limited medical personnel and equipment. To the best of our knowledge, only a few studies were published in the literature evaluating the addition of SNB to ACB and PIA in pain management after TKA. Kampitak et al. reported that SNB utilizing 0.25% levobupivacaine (15 ml) with an ACB and PIA decreased morphine consumption. Nevertheless, the SNB group demonstrated a statistical difference in its degree of pain reduction relative to an obturator-nerve-block group [20]. The other study from the same group of authors also compared SNB (0.25% levobupivacaine 15 ml) to the interspace between the popliteal artery and the capsule of the posterior knee (IPACK block) after TKA [16]. They reported a higher incidence of a motor blockade in the SNB group than IPACK, but the SNB group had a lower incidence of posterior knee pain. In both studies, SNBs were performed with a higher concentration of local anesthetic and included postoperative NSAIDs in the protocol. In comparison, our study used a low-dose SNB (20 ml of 0.125% bupivacaine) and dexamethasone (5 mg) combined with PIA and ACB and revealed the effectiveness for post-TKA pain control without motor weakness. Despite no systemic NSAIDs given, low-dose SNB was still significantly decreased pain scores at rest, on motion, and anterior knee up to 18–24 h following TKA.

Although we primarily hypothesized that pain would be alleviated in the SNB group due to the analgesia in the sciatic nerve distribution, mainly to reduce posterior knee pain. However, the median posterior knee pain score was reduced only at 12 h. One of the reasons would be our intention to use a low concentration of bupivacaine (0.125%) for SNBs to limit local anesthetic doses. With this concentration, the patients might not be able to characterize the specific area of pain, particularly if the pain was mild. Secondly, only 5 (20%) patients (1 in the SNB and 4 in the control group) reported moderate-severe posterior knee pain. Due to the small sample size, it might be challenging to demonstrate the statistical difference in posterior knee pain. The incidence of moderate-severe posterior knee pain in our study also correlates to the previous studies by Gi et al. and Kampitak et al. The authors reported that 10–30% of the patients underwent TKA with a femoral nerve block and PIA had posterior knee pain [16, 21]. Therefore, the benefit from adding SNB to adductor canal block and PIA may not be solely explained by posterior knee pain reduction.

We also added dexamethasone 5 mg to the SNB, while the control group patients did not receive dexamethasone. It has been well-demonstrated that dexamethasone is an effective adjunct to other multimodal analgesia techniques for postoperative pain control in TKA, regardless of the routes of administration [18, 22–24]. Although a meta-analysis by De Oliveira et al. had reported that low dose dexamethasone (< 0.1 mg/kg) did not provide opioid-sparing effect or analgesia during the early postoperative period (< 4 h), however, dexamethasone significantly reduced late pain scores (24 h) both at rest and on movement, even at low dose [24]. Since we compared pain scores at 6–24 h postoperatively, dexamethasone given in the SNB injections to prolong the duration of the block may potentially be a confounder in our study and caused better pain relief in the SNB group compared to the control.

Although the mean 24-h morphine consumption was statistically lower in the SNB group (1.96 ± 2 mg Vs. 3.80 ± 2.48 mg), the slight difference may have modest clinical effects. The overall morphine consumption in our study was relatively lower than that reported from the earlier studies in patients with TKA. The reason might be that the patient-controlled analgesia was not cooperated in our protocol for morphine administration. Additionally, in older studies, 24-h morphine consumption and pain score were relatively high because multimodal analgesia combined with ultrasound-guided peripheral nerve block technique had not yet been a standard of care [19, 25]. Among newer studies in which multimodal analgesia was utilized with improvement in peripheral nerve block technique and PIA, average total doses of 24-h morphine consumption also ranged only between 2 and 10 mg [9, 16, 26]. In our study, overall 24-h morphine consumption was considered low in both groups, and the difference was only approximately 2 mg which may not have significant clinical effects. The combination of ACB and PIA had already provided substantial pain control for most patients even without the use of NSAIDs. Therefore, the further opioid-sparing benefit of SNB was not well determined.

The risks of bupivacaine toxicity when combining several blocks must be taken into account. We limited the total dose of bupivacaine for the peripheral nerve blocks not to exceed 1.7 mg/kg (75 mg for 45 kg patient). Also, 100 mg of PIA was a low range commonly used in other studies, 100–400 mg, due to minimal absorption at this injection site and was considered safe by several studies [16, 27, 28]. Additionally, the duration between performing the peripheral nerve blocks and the PIA was about 90–120 min, resulting in a mismatch of the peak plasma concentration of bupivacaine. The peak plasma concentration of peripheral nerve block is at 60 min [29–31], while the peak plasma concentration of PIA is at 24–48 h [32]. Previous studies showed that the total plasma concentrations of the maximum recommended doses of bupivacaine used in the peripheral nerve block and the PIA remained below half of the described toxicity thresholds. The margin of safety was probably much broader than current standards or the manufacturer’s recommended dose [29–32]. However, all patients were closely monitored for the local anesthetic systemic toxicity (LAST) because of high doses of the local anesthetic in this study, especially in low body weight patients.

There are some limitations to this study. First, this was a single-center study using a small sample size, which had led to limitations for the analysis of the difference in posterior knee pain. Secondly, we did not use patient-controlled analgesia for administering morphine postoperatively, therefore, it might underrepresent the 24-h morphine dose. Third, NSAIDs was still used in this study as a single dose of ketorolac in PIA. Only systemic NSAIDs was omitted. Finally, dexamethasone added into the SNB for an extended duration of analgesia may be a potential confounder for better pain control in the SNB group.

## Conclusions

For patients susceptible to the adverse effects of NSAIDs, adding a low concentration of SNB combined with dexamethasone is an effective adjunctive technique to ACB and PIA for acute postoperative pain control in TKA. It offers benefits in early postoperative pain control (especially pain on movement) without a significant motor blockade.

## Data Availability

The datasets used and/or analyzed during the current study are available from the corresponding author on reasonable request.

## Abbreviations

SNB: Sciatic nerve block
TKA: Total knee arthroplasty
NSAID: Non-steroidal anti-inflammatory drug
ACB: Adductor canal block
PIA: Periarticular infiltration analgesia
SD: Standard deviation
IQR: Interquartile range
BMI: Body mass index
CKD: Chronic kidney disease
Cr: Creatinine
DM: Diabetes mellitus
eGFR: Glomerular filtration rate
HT: Hypertension
IHD: Ischemic heart disease
NRS: Numeric rating scale
COX-2 inhibitor: Cyclooxygenase-2 inhibitor
IPACK: Infiltration of the local anesthetic between the popliteal artery and capsule of the knee
LAST: Local anesthetic systemic toxicity
